# Research Progress in Pharmacological Effects and Mechanisms of *Angelica sinensis* against Cardiovascular and Cerebrovascular Diseases

**DOI:** 10.3390/molecules29092100

**Published:** 2024-05-02

**Authors:** Linlin Chen, Bei Fan, Fengzhong Wang, Yang Song, Xizhi Wang, Ying Meng, Yumin Chen, Qing Xia, Jing Sun

**Affiliations:** 1 School of Management, Liaoning University of International Business and Economics, Dalian 116052, China; chenlinlin@luibe.edu.cn (L.C.); songyang@luibe.edu.cn (Y.S.); wangxizhi@luibe.edu.cn (X.W.); mengying@luibe.edu.cn (Y.M.); chenyumin@luibe.edu.cn (Y.C.); 2 Risk Assessment Laboratory of Agricultural Products Processing Quality and Safety, Key Laboratory of Agricultural Products Quality and Safety Collection, Storage and Transportation Control, Ministry of Agriculture and Rural Affairs, Institute of Food Science and Technology, Chinese Academy of Agricultural Sciences, Beijing 100193, China; fanbei517@163.com (B.F.); wangfengzhong@caas.cn (F.W.); 3 Biology Institute, Qilu University of Technology (Shandong Academy of Sciences), Jinan 250013, China

**Keywords:** *Angelica sinensis*, medicinal and edible values, cardiovascular and cerebrovascular diseases, volatile oil, polysaccharide

## Abstract

*Angelica sinensis* (Oliv.) Diels (*A. sinensis*) is a medicinal and edible values substance, which could promote blood circulation and enrich blood. It possesses rich chemical components and nutrients, which have significant therapeutic effects on cardiovascular and cerebrovascular diseases. It is commonly used for the prevention and treatment of cardiovascular and cerebrovascular diseases in the elderly, especially in improving ischemic damage to the heart and brain, protecting vascular cells, and regulating inflammatory reactions. This article reviews the main pharmacological effects and clinical research of *A. sinensis* on cardiovascular and cerebrovascular diseases in recent years, explores the effect of its chemical components on cardiovascular and cerebrovascular diseases by regulating the expression of functional proteins and inhibiting inflammation, anti-apoptosis, and antioxidant mechanisms. It provides a reference for further research on *A. sinensis* and the development of related drugs. It provides a new reference direction for the in-depth research and application of *A. sinensis* in the prevention, improvement, and treatment of cardiovascular and cerebrovascular diseases.

## 1. Introduction

As a typical chronic disease of the elderly, cardiovascular and cerebrovascular diseases have always been one of the main causes of human death worldwide [1,2], with the characteristics of high prevalence, high morbidity, and high mortality [3], and accompanied by complex and diverse psychological diseases. Cardiovascular and cerebrovascular diseases are a group of diseases caused by ischemic or hemorrhagic changes in cardiovascular, cerebrovascular, and systemic tissues caused by atherosclerosis, hyperlipidemia, hypertension, and other risk factors [4,5]. China has a large population. The problem of population aging came earlier than people expected. With the aggravation of population aging, bad eating habits, and irregular work and rest, cardiovascular and cerebrovascular diseases have become a major health problem for the Chinese people [6]. According to some statistics, elderly people over 60 years old account for about 18% of the total population in China, and more than 50% of them suffer from cardiovascular and cerebrovascular diseases. In 2019, the number of cardiovascular and cerebrovascular diseases in China has reached 330 million, and the annual death number has exceeded 4 million, ranking first [7]. At present, cardiovascular and cerebrovascular diseases are not exclusive terms for the elderly, and the risk of the diseases is slowly approaching for the young. If not prevented and treated, it will seriously hinder the smooth progress of the construction of “Healthy China 2030” and seriously endanger people’s life and health.

*Angelica sinensis* is the dried root of the umbrella-family plant *Angelica sinensis* (Oliv.) Diels (*A. sinensis*). It is one of the commonly used food and medicinal substances in China and has a long history of food and drug use [8,9]. As a kind of blood-promoting medicine, *A. sinensis* has the traditional effect of invigorating blood circulation, regulating menstruation, relieving pain, moisturizing the intestines, and relieving constipation [10,11]. There are about 90 species of classified plants in the world, which are mainly distributed in the north temperate zone, and 45 species are distributed in our country [12]. *A. sinensis* was first recorded in Shennong’s Classic of Materia Medica, “*A. sinensis* tastes warm and non-toxic”. It has been recorded in many ancient and modern books [10,13]. It can be seen that *A. sinensis* has a long history of use and is known as “the holy medicine of blood family” and “the holy medicine of gynecology” among the people [8]. In China, *A. sinensis* is not only a traditional Chinese medicine, but also a widely used spice, condiment, and nutritional agent in the traditional diet. *A. sinensis* is rich in nutrients, such as organic acids, volatile oils, amino acids, etc. [14]. So, *A. sinensis* is used as a spice and nutritional supplement in some European, American, and African countries [8,15,16].

**Figure 1:**
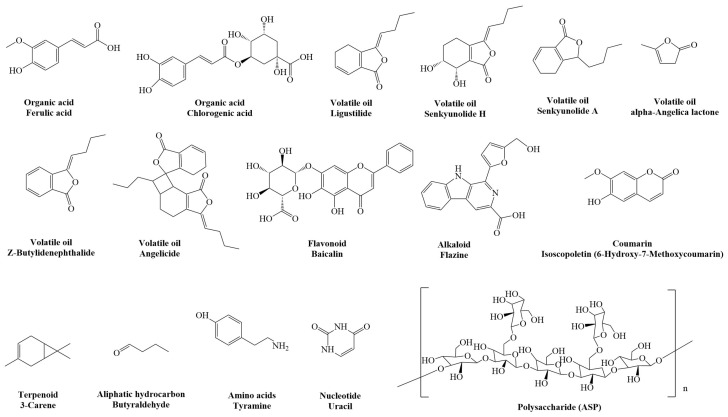
The main chemical constituents of *A. sinensis* and their representative compounds.

At the same time, with the development of modern analytical technology, it has been found that volatile oil, organic acids, phenolic acids, flavonoids, coumarin, polysaccharides, amino acids, and rich nutrients are important active components of *A. sinensis*. Modern pharmacological studies also show that *A. sinensis* plays a role in improving hemorheology and has good anti-Alzheimer’s disease, anti-tumor, antioxidation, anti-inflammatory, and analgesic activities [17,18,19,20,21]. In addition, *A. sinensis* has been shown to have significant effects on cardiovascular and cerebrovascular diseases, such as anti-atherosclerosis, regulation of blood glucose and blood lipids, inhibition of thrombosis, and improvement of cognitive impairment, and these effects are inseparable from the active ingredients of *A. sinensis*. The protective effects of *A. sinensis* on cardiovascular and cerebrovascular diseases have been widely studied. Firstly, we consulted the databases of domestic and foreign literature and searched the literature using *A. sinensis* as the keyword, such as Web of Science, Science Direct, China National Knowledge Infrastructure (CNKI), and so on. And more than 20,000 domestic and foreign articles related to *A. sinensis* in from the last 10 years were collected. Subsequently, we used “chemical composition” and “cardiovascular and cerebrovascular diseases (cardiovascular, cerebrovascular, vascular, etc.)” as keywords to screen the literature in the past decade. We found that *A. sinensis* has numerous chemical components and has significant preventive and therapeutic effects on cardiovascular and cerebrovascular diseases. Then, software Chemdraw 19.0 was used to map some important compounds in *A. sinensis*, Figure 1 and other figures. Finally, the pharmacological effects of *A. sinensis* on cardiovascular and cerebrovascular diseases were classified into cardiovascular diseases, cerebrovascular diseases, and other vascular diseases in this review, and the research progress of the pharmacological effect of *A. sinensis* on cardiovascular and cerebrovascular diseases in the past decade was summarized. Therefore, this article aims to provide a new reference direction for the in-depth research of *A. sinensis* in the prevention, improvement, and treatment of cardiovascular and cerebrovascular diseases, and to provide new ideas for the development and utilization of traditional Chinese medicine in the field of elderly care.

## 2. Pharmacological Effects and Mechanisms of *A. sinensis* on Cardiovascular Diseases

### 2.1. Influence of Myocardial Ischemic Injury

Ischemic myocardial injury is caused by a sharp decrease or interruption of blood supply to the coronary arteries, resulting in significant damage to the myocardium [22]. When myocardial cells are damaged and then reperfused, they enter a state of hypoxia or re-oxygenation, leading to severe damage to mitochondria and cell membranes. This can cause abnormal cell membrane permeability, leakage of myocardial markers, and an increased risk of heart failure [23,24]. Ultimately, it can result in myocardial ischemia–reperfusion injury (MIRI), which affects cardiovascular function in humans. Ye et al. [25] found through the MIRI model that the polysaccharides of *A. sinensis* can improve MIRI in rats and protect myocardial function in both in vivo and in vitro ischemia reperfusion models. After treatment with polysaccharides of *A. sinensis*, the activity of rat myocardial cells increased, the secretion of inflammatory factors decreased, the expression of TLR4/NF-*κ*B signaling pathway-related proteins decreased, and the expression of apoptosis-related proteins was down-regulated [25]. At the same time, polysaccharides of *A. sinensis* can also inhibit the up-regulation of apoptosis factor Bcl-2 expression and promote the down-regulation of apoptosis factor Bax expression [26]. The mechanism by which polysaccharides of *A. sinensis* improve MIRI may be through inhibiting the TLR4/NF-*κ*B pathway activation, alleviating inflammation, reducing cell apoptosis and thereby relieving MIRI. Meanwhile, it was also found that the main component ligustilide, in the volatile oil of *A. sinensis*, has the same inhibitory effect on the My D88/TLR4/NF-*κ*B signaling pathway, inhibiting the expression of inflammation factors such as NO, IL-6, and TNF-*α*, and is used for an inhibitory effect on MIRI [27].

**Figure 2:**
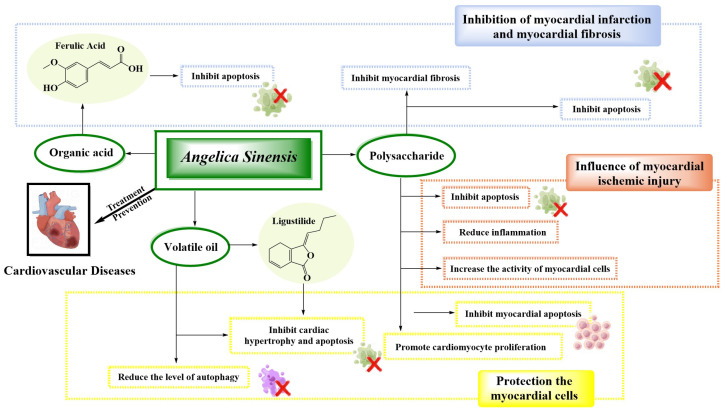
Mechanisms of *A. sinensis* and its compounds in the treatment of cardiovascular diseases.

The polysaccharides in *A. sinensis* are one of the most important components for improving MIRI. Polysaccharides and ligustilide have certain similarities in improving MIRI, mainly by reducing the expression of inflammatory factors, inhibiting the occurrence of the inflammatory response, and inhibiting the activation of the TLR4/NF-*κ*B signaling pathway and the expression of related proteins (Figure 2 and Table 1). However, *A. sinensis* polysaccharides can also be used to inhibit the increase of Bcl-2 expression, promote a decrease in Bax expression, inhibit myocardial cell apoptosis, improve and alleviate myocardial ischemia injury, improve myocardial function, and prevent the occurrence of cardiovascular disease. This provides an effective basis for the clinical application of polysaccharides of *A. sinensis* in cardiovascular and cerebrovascular diseases (Table 1).

### 2.2. Protection of Myocardial Cells

Mature cardiomyocytes are highly differentiated terminal cells, which are closely related to cardiovascular diseases and affect the occurrence and change of cardiovascular diseases. Cui et al. [28] found that the blood glucose and lipid levels of diabetic KK-Ay mice could be regulated by polysaccharides of *A. sinensis*, mainly by down-regulating the expression level of cleaved-caspase-3, increasing the expression level of Bcl-2, and inhibiting the endoplasmic reticulum stress-IRE1/ASK1/JNK signaling pathway and other pathways. The results showed that the purpose of inhibiting the apoptosis of cardiac myocytes could be achieved through the above pathways, so as to play a protective role for myocardial cells. Some scholars have found that the protective effect of polysaccharides in *A. sinensis* on cardiomyocytes plays a role mainly by up-regulating the expression of miR-4701-3p, down-regulating the expression of miR-22, promoting the proliferation of H9c2 cells, inhibiting the apoptosis of cardiac myocytes, and reducing the number of damaged cardiac myocytes [29,30]. The myocardial cell apoptosis and hypertrophy induced by angiotensin II (Ang II) could be significantly ameliorated by *A. sinensis* and its volatile oil. The adverse effects induced by Ang II can be effectively antagonized, and the apoptosis of vascular endothelial cells can be effectively inhibited by *A. sinensis*. However, a good calcium blocking effect is possessed by the volatile oil of *A. sinensis*, which could antagonize Ca^2+^ overload and inhibit the protein expression of calcineurin (CaN) and T-type calcium channel subtypes (Cav3.1, Cav3.2) by Ang II, and the effect was more obvious at high doses. Therefore, *A. sinensis* and its volatile oil can significantly inhibit cardiomyocyte hypertrophy and apoptosis and protect cardiac myocytes [31,32]. Pu et al. [33] found that the volatile oil of *A. sinensis* could be used to regulate the expression of autophagy-related proteins (Beclin-1, LC3II, LC3I, and p62) to reduce the level of autophagy in cardiac myocytes damaged by hypoxia and oxygen enrichment, thus playing a protective role for cardiac myocytes.

This significant myocardial protective function is one of the biological activities of the volatile oil and polysaccharides in *A. sinensis*. The protective effect of *A. sinensis* polysaccharides on myocardial cells is closely related to regulating the expression level of related proteins, regulating the endoplasmic reticulum stress IRE1/ASK1/JNK signaling pathway, promoting the proliferation of related myocardial cells, and inhibiting the apoptosis of myocardial cells (Figure 2 and Table 1). However, the protection of myocardial cells, reduction of myocardial cell damage, and prevention of cardiovascular diseases are the main effects of *A. sinensis* and its volatile oil. Its biological activity is mainly used for improving the induction effect of angiotensin II, inhibiting Ca^2+^ overload, inhibiting the apoptosis of vascular endothelial cells, and regulating the level of autophagy-related proteins (Table 1).

### 2.3. Inhibition of Myocardial Infarction and Myocardial Fibrosis

Myocardial infarction (MI) is regarded as one of the leading causes of death and disability worldwide. The common pathological mechanism of many cardiovascular diseases is myocardial fibrosis, and the occurrence of cardiovascular diseases is significantly affected by MI and myocardial fibrosis [68]. Chen et al. [34] found that *A. sinensis* had a good protective effect against MI. This effect is closely related to reducing the release of creative kinase (CK) and lactate dehydrogenase (LDH) in cardiomyocytes, enhancing the expression of myocardial apoptosis-related protein Bc1-2, and reducing the expression of Bax. Zhao et al. [35] found that *A. sinensis* can be used to significantly improve the occurrence of myocardial fibrosis after MI, effectively prevent and treat myocardial fibrosis after MI, and improve cardiac function through in vivo rat experiments. The results showed that the effect of *A. sinensis* on myocardial fibrosis after MI was related to the inhibition of macrophage infiltration and the down-regulation of TGF-*β*1 expression, blocking the occurrence of fibrosis, and effectively alleviating the excessive deposition of reactive collagen in the non-infarction area after MI. At the same time, *A. sinensis* and its polysaccharides can be used to reduce and prevent MI and myocardial fibrosis, mainly by down-regulating miR-22 expression in hypoxia-treated H9c2 cells, and reducing attenuated hypoxia-induced H9c2 cell injury [36], enhancing antioxidant capacity in vivo, reducing the expression level of pro-inflammatory factors, regulating the expression of the AMPK-PGC1α pathway and the expression levels of Bcl-2 and Bax, and decreasing endoplasmic reticulum (ER) stress-induced cell death both in vitro and in vivo in ischemia injury rats [37], and increasing the activity of superoxide dismutase (SOD) and glutathione peroxidase (GSH-Px) and decreasing the level of malondialdehyde (MDA), tissue endogenous hydrogen peroxide (H_2_O_2_), and reactive oxygen species (ROS) [38]. Taken collectively, *A. sinensis* and its polysaccharides were used to inhibit the apoptosis of cardiac myocytes and to prevent, improve, and reverse early ventricular remodeling after MI and acute myocardial infarction [37,38]. *A. sinensis* and its polysaccharides played a protective role in myocardial infarction and myocardial fibrosis, improved cardiac function, and prevented cardiovascular diseases by these pathways. Li et al. [39] found that ferulic acid can be used to protect cardiac myocytes from apoptosis by enhancing autophagy and, at the same time, can be used to decrease ROS production, inhibit caspase3 activation, decrease the apoptotic cell number, reduce myocardial infarct size, and improve cardiac function in an in vivo mouse MI model.

*A. sinensis* has a good effect on improving MI and myocardial fibrosis, and its polysaccharides are one of the main chemical components that play a role in improving MI and myocardial fibrosis (Figure 2). The mechanism by which *A. sinensis* improves MI and myocardial fibrosis is related to reducing the release of related enzymes in myocardial cells, regulating the expression level of myocardial apoptosis-related proteins, inhibiting the activity of macrophages, and blocking the occurrence of fibrosis. The polysaccharide is used to inhibit myocardial cell apoptosis by inhibiting the infiltration and accumulation of macrophages, enhancing antioxidant capacity in vivo, reducing the level of pro-inflammatory factors, regulating the expression level of related proteins, and so on (Figure 2 and Table 1). However, the ferulic acid in *A. sinensis* is mainly used to protect cardiac myocytes from apoptosis by enhancing autophagy, inhibiting MI, and preventing cardiovascular diseases. Taken collectively, *A. sinensis* plays a protective role against myocardial infarction and myocardial fibrosis, improves cardiac function, and prevents cardiovascular diseases (Table 1).

## 3. Pharmacological Effects and Mechanisms of *A. sinensis* on Cerebrovascular Diseases

### 3.1. Influence of Cerebral Ischemic Injury

Ischemic cerebral vascular disease (ICVD), a disease with a high disability rate, is a kind of local oxygen supply and energy metabolism imbalance caused by insufficient blood supply of brain tissue, which eventually leads to brain tissue damage and irreversible necrosis of neurons [69,70]. Dai et al. [69] studied the effect of *A. sinensis* polysaccharides on inflammatory factors through a rat model of cerebral ischemia reperfusion injury. The results showed that when the dosage of polysaccharides was 60 mg/kg, the levels of IL-1β, TNF-α, and NF-κB were effectively reduced, and the inflammatory reaction in the brain tissue of ischemia reperfusion was alleviated, which was helpful for improving cerebral ischemia injury and restoring neurological function [69]. In addition, polysaccharides of *A. sinensis* can also be used to reduce the level of oxidative stress in brain tissue to protect against cerebral ischemia reperfusion injury [40]. Li et al. [41,42] found that polysaccharides of *A. sinensis* with low molecular weight can be applied to effectively inhibit the apoptosis of hippocampal neurons by up-regulating the expression of Bcl-2 and down-regulating the expression of Bax and Cyto C, significantly promoting angiogenesis in brain tissue and reducing ischemic injury, so as to alleviate cerebral ischemia reperfusion injury. Studies have found that the volatile oil of *A. sinensis* can be used to significantly improve blood hypercoagulation and blood viscosity, enhance the viability of cells, and improve hemorheology [43]. In addition, the anti-cerebral ischemia effect of *A. sinensis* and ligustilide has been applied mainly by activating the phosphorylated protein kinase B (Akt)/mammalian target of rapamycin (mTOR) signal transduction pathway and regulating the expression of autophagy protein (p-Akt, p-m TOR, and p-P70S6K) [44,45]. Ligustilide in *A. sinensis* can be used to improve cerebral ischemic injury, mainly by effectively inhibiting the expression of Prx5 and Prx6 and its downstream TLR4/NF-κB signal transduction, reducing the expression levels of inflammatory factors IL-6 and IL-8, inhibiting cell apoptosis (up-regulating Bcl-2, down-regulating Bax and caspase), and inhibiting oxidative stress [46,47,48].

**Figure 3:**
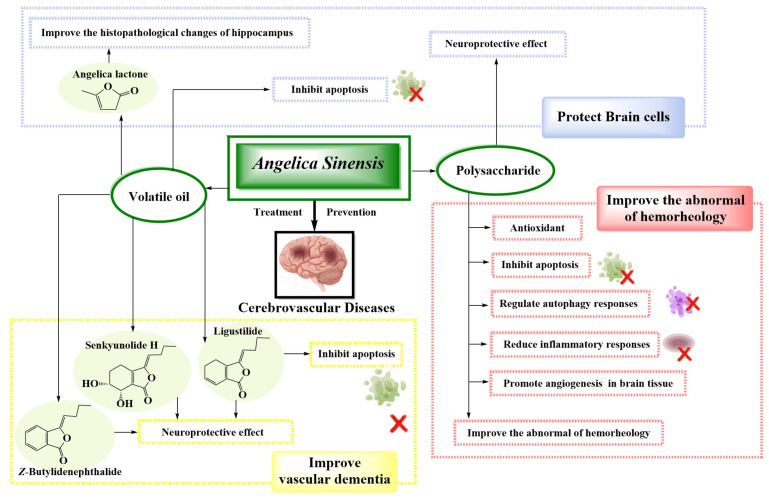
Mechanisms of *A. sinensis* and its compounds in the treatment of cerebrovascular diseases.

The polysaccharides and volatile oil of *A. sinensis* are the main components that improve cerebral ischemic injury. The significant improvement of hemorheology is dependent on the blood-activating effect of *A. sinensis*, which can effectively prevent the occurrence of cerebrovascular diseases. Polysaccharides of *A. sinensis* are used to protect from cerebral ischemic injury and prevent the occurrence of cerebrovascular diseases by inhibiting inflammatory reactions, reducing the level of oxidative stress in brain tissue, inhibiting nerve cell apoptosis, and promoting cerebral angiogenesis. However, the volatile oil of *A. sinensis* has been used to regulate abnormal hemorheology indicators, inhibit apoptosis of neurocytes by activating the AKT/mTOR signaling pathway and other pathways, inhibit thrombosis, regulate autophagy responses, inhibit inflammatory responses, and exert antioxidant activity to play an anti-cerebral ischemia effect, and effectively prevent cerebrovascular diseases (Figure 3 and Table 1).

### 3.2. Improvement in Vascular Dementia

Vascular dementia (VD), a dementia syndrome, is considered to be caused by brain dysfunction caused by various cerebrovascular diseases, with learning and memory impairment as the main symptoms [49,71,72]. However, cerebral ischemia is regarded as one of the main causes of VD. Wu Guotai et al. [50] found that the volatile oil of *A. sinensis* can be used to promote the expression of Bcl-2 protein and inhibit the expression of Bax protein and the apoptosis of nerve cells. The results show that the volatile oil of *A. sinensis* can be used to enhance learning and memory abilities, improve cognitive impairment, relieve senility and vascular dementia, and play a protective role in the brain. Methods were utilized by Fan et al. [49], such as network pharmacology, molecular docking, and experimental verification, to discover that the core active ingredients of E-ligustilide, senkyunolide H, and *Z*-butylidenephthalide in *A. sinensis* can be effectively applied to relieve cognitive dysfunction in vascular dementia. Significant neuroprotective effects are possessed by ligustilide and *Z*-butylidenephthalide. Additionally, ligustilide can also be used to alleviate damage to hippocampal neurons after cerebral ischemia–reperfusion by activating the PINK1/Parkin pathway-mediated mitophagy [51,52].

Cerebrovascular dementia is a type of dementia in the elderly. Dong quai has significant therapeutic effects in treating dementia in the elderly, and the volatile oil of *A. sinensis* is one of the main components in preventing cerebrovascular dementia. The active ingredients, such as ligustilide, senkyunolide, and *Z*-butylidenephthalide, in the volatile oil of *A. sinensis* play a role in slowing down cognitive function impairment and preventing cerebrovascular dementia in the elderly (Figure 3 and Table 1). Its mechanism is mainly related to regulating the expression levels of relevant proteins, activating related pathways to mediate mitochondrial autophagy function, and inhibiting neuronal apoptosis.

### 3.3. Protection of Brain Cells

Che et al. [53] found that polysaccharides of *A. sinensis* can be used to inhibit endoplasmic reticulum stress (down-regulating the expression of GRP78, CHOP, and Caspase-12), reduce neuronal apoptosis, and improve learning and memory abilities in rats, which was played a protective role in the pathological process of Alzheimer’s disease. Zhu et al. [54] found that *A. sinensis* volatile oil and its core components ((−)-(*S*)-1-(4-hydroxy-3-methoxyphenyl)-ethyl methyl ether, pentanone, and monomethyl phthalate) were exerting neuroprotective effects by significantly increasing cell survival rate at a certain concentration, reducing the level of LDH in cells, inhibiting the apoptosis of PC12 cells, improving damaged cell permeability, and inhibiting neuronal cell apoptosis. There is research that shows that angelica lactone can be used to effectively lower the levels of malondialdehyde (MDA), tumor necrosis factor-α (TNF-*α*), and interleukin-1β (IL-1*β*) in brain tissues, as well as the level of Bax in the hippocampus. It can also increase the activity of superoxide dismutase (SOD) in brain tissues and the level of Bcl-2 in the hippocampus, improve the histopathological changes of rats’ hippocampus, and exert a protective effect on brain neurons against ischemic vertigo [55].

Brain cells are composed of various cells in the brain. A significant regulatory effect on the nervous system is owned by *A. sinensis*, and a good protective effect on brain cells is owned by the polysaccharides and volatile oil in *A. sinensis*. The mechanism by which the polysaccharides protect brain cells is closely related to the inhibition of endoplasmic reticulum stress and the reduction of neuronal apoptosis (Figure 3 and Table 1). However, the volatile oil of *A. sinensis* has been used to improve the survival rate of brain nerve cells, relieve hippocampal tissue damage, and reduce the occurrence of cerebrovascular diseases, mainly by reducing the activity of related enzymes in brain cells, reducing the expression level of related inflammatory factors, and inhibiting cell apoptosis (Table 1).

## 4. Pharmacological Effects and Mechanisms of *A. sinensis* on Other Vascular Diseases

### 4.1. Regulation of Blood Pressure and Blood Lipids

Qu et al. [56] found through in vivo experiments on rats that volatile oil has a certain hepatoprotective effect and can be used effectively to improve spontaneous hypertension and regulate blood lipids. The experiment showed that the mechanism of volatile oil regulating blood pressure and blood lipids is closely related to up-regulating the expression levels of the Tnfaip8l2 and Ahsg genes, and inhibiting atherosclerosis. Jiang et al. [57,58] analyzed the effects of *A. sinensis* on the PI3K/Akt/eNOS signaling pathway that were related to the vascular endothelial system in hypertension through animal experiments, and they found that the volatile oil of *A. sinensis* had a good anti-hypertensive effect by regulating the expression levels of PI3K/Akt/eNOS proteins, which are closely related to protecting vascular endothelial cells and smooth muscle. The research showed that polysaccharides can be used effectively to increase the activities of SOD and GSH-Px and reduce levels of MDA and endogenous H_2_O_2_ and reactive ROS, through inhibiting myocardial fibrosis and myocardial apoptosis and alleviating oxidative stress. Through these pathways, it is used to prevent hypertensive heart disease, thus effectively preventing the occurrence of cardiovascular and cerebrovascular diseases [39].

**Figure 4:**
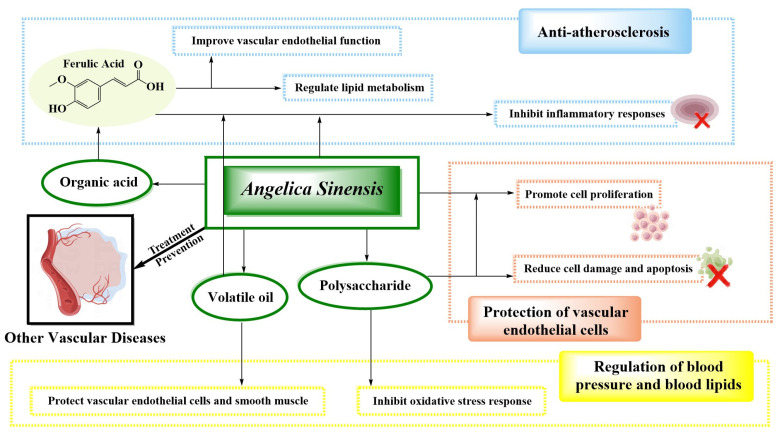
Mechanisms of *A. sinensis* and its compounds in the treatment of other vascular diseases.

Both volatile oil and polysaccharides in *A. sinensis* have significant effects in regulating blood pressure and blood lipid levels (Figure 4 and Table 1). Volatile oil is used mainly to inhibit atherosclerosis by regulating the expression of lipid metabolism-related genes and protein expression in the endothelial system-related signaling pathways, protecting endothelial cells and smooth muscle cells, and regulating blood pressure and blood lipid levels. Polysaccharides play a role in regulating blood pressure and blood lipids closely related to their antioxidant effects, mainly by reducing oxidative stress response, inhibiting myocardial fibrosis, inhibiting myocardial apoptosis, and effectively preventing and treating hypertension and hyperlipidemia (Table 1).

### 4.2. Anti-Atherosclerosis

Atherosclerosis (AS), one of the important pathological bases for the occurrence of cardiovascular and cerebrovascular diseases, is mainly closely related to inflammation, oxidative stress, high homocysteine levels, and abnormal blood lipid levels [73]. Fang et al. [59] discovered that 17 chemical components in *A. sinensis* can directly or indirectly act on 26 related core drug targets to exert anti-atherosclerotic effects. These drug targets involve the regulation of 10 related pathways, including cholesterol trans-membrane transport, cholesterol ester metabolism, inflammatory response, lipid oxidation modification, and the expression of related active genes. At the same time, it was also discovered that some organic acids and volatile oils in *A. sinensis* can activate receptors through peroxisomes to regulate inflammation and lipid metabolism pathways, thus combating the occurrence of AS. Wang et al. [60] concluded that ferulic acid has a good clinical effect on intervening in coronary atherosclerotic heart disease angina in the elderly. Its mechanism of action is mainly related to clearing excess free radicals in the body, reducing membrane lipid peroxidation, and increasing the activity of antioxidant enzymes. At the same time, sodium ferulic acid was also used to reduce plasma endothelin levels and increase NO levels, thereby improving vascular endothelial function and further improving the clinical symptoms of coronary atherosclerotic heart disease angina. The research shows that the volatile oil, polysaccharides, organic acids, and flavonoids in *A. sinensis* mainly played a role in treating AS by inhibiting the proliferation of vascular smooth muscle cells, anti-platelet aggregation, inhibition of the inflammatory reactions, antioxidant stress response, the regulation of blood lipids, the regulation of autophagy activity, etc., and focus on several classic pathways such as MAPKs, TLR4/NF-*κ*B, Nrf2/HO-1, and PI3K/Akt/mTOR [61,62,63,64,65]. One of these is the NF-*κ*B signaling pathway, being considered as a classic inflammatory signaling pathway. Its mediated inflammatory response is closely linked to processes such as vascular smooth muscle cell proliferation and lipid regulation [61].

Volatile oils, polysaccharides, organic acids, and flavonoids are the main active ingredients of *A. sinensis* in exerting anti-atherosclerotic effects, and these components can mediate multiple signaling pathways to improve hemodynamics and vascular endothelial function (Table 1). In conclusion, the anti-atherosclerotic effects of *A. sinensis* are closely related to the inhibition of vascular smooth muscle cell proliferation, anti-platelet aggregation, inhibition of the inflammatory response, antioxidant stress response, regulation of blood lipids, regulation of autophagy activity, and so on (Figure 4 and Table 1).

### 4.3. Protection of Vascular Endothelial Cells

Studies have shown that the level of NO and the activity of endothelial nitric oxide synthase in human vascular endothelial cells can be increased by polysaccharides, thereby reducing the damage of vascular endothelial cells and apoptosis induced by oxidized low-density lipoprotein, and the expression of vascular endothelial growth factor can also be significantly enhanced [66], and volatile oil exerted strong antioxidant capabilities and protective effects on endothelial cells and was used for significantly increasing the survival rate of endothelial cells damaged by hydrogen peroxide, reducing mortality, increasing the activity of SOD and NO in cells, lowering levels of LDH and MDA, alleviating oxidative damage to vascular endothelial cells, and restoring endothelial cell proliferation. Chen et al. [67] found that polysaccharides had protective effects on bone marrow stromal cells and the vascular endothelial growth factor of mice damaged by radiation through animal experiments. The results showed that polysaccharides could enhance the expression of vascular endothelial growth factor, increase the proliferation ability of bone marrow-derived mesenchymal stem cells, promote the proliferation of vascular endothelial cells, and form new blood vessels in bone marrow. Through those pathways, the nutrition for the proliferation, differentiation, and maturation of hematopoietic stem cells were provided, their apoptosis was reduced, and the recovery of hematopoietic function was accelerated further.

The volatile oil and polysaccharides of *A. sinensis* are the main chemical components that protect vascular endothelial cells. Volatile oil plays a protective role for vascular endothelial cells by improving the activities of superoxide dismutase and nitric oxide (Figure 4 and Table 1). The mechanism of polysaccharides is mainly to regulate the expression of nitric oxide and nitric oxide synthase in human vascular endothelial cells, enhance the expression of endothelial growth factor, and, thus, reduce the damage of vascular endothelial cells (Table 1).

## 5. Conclusions and Research Outlook

According to relevant reports, it is known that the incidence of cardiovascular and cerebrovascular diseases in our country is relatively high, with the main patient group being elderly people. As one of the traditional Chinese medicinal materials for tonifying deficiency, *A. sinensis* not only has unique traditional medicinal effects and modern pharmacological activities, but also has rich nutrients and edible benefits, which has a good effect in the prevention and treatment of cardiovascular and cerebrovascular diseases in the elderly. It is not difficult to find that the organic acids (ferulic acid), volatile oil (ligustilide, senkyunolide H, and butylidenephthalide), and polysaccharides in *A. sinensis* are the main chemical components that play a role in preventing cardiovascular and cerebrovascular diseases. Its protective effects against cardiovascular and cerebrovascular diseases are mainly related to regulating the expression levels of relevant enzymes, proteins, and genes, inhibiting or activating the autophagic function of cells, improving abnormal blood rheology, regulating oxidative stress response, reducing inflammatory reactions, and suppressing related cell apoptosis pathways.

Currently, products of *A. sinensis* are mainly distributed in the fields of pharmaceuticals, food, health products, and cosmetics on the market, widely used by middle-aged and elderly people as well as female groups, with about 600 products being involved. Therefore, in the application, different products of *A. sinensis* should be developed for the different needs of middle-aged and elderly people in the prevention of cardiovascular and cerebrovascular diseases so as to achieve better application effects in the field of traditional Chinese medicinal health care for the elderly. Although in clinical practice *A. sinensis* is commonly used for the prevention and treatment of cardiovascular and cerebrovascular diseases such as elderly dementia, myocardial infarction, and brain injury, the specific mechanisms by which *A. sinensis* exerts its therapeutic effects is still not completely understood, limiting its practical applications in clinical and daily life. Therefore, additional research is needed to explore the mechanism of *A. sinensis* in preventing cardiovascular and cerebrovascular diseases. Targeted development and application of *A. sinensis* for the elderly should be implemented to fully utilize its significant role in preventing these diseases in this population. This will provide theoretical support for the integration of traditional Chinese medicine and elderly care industries, further advancing the development of a combined retirement model that promotes the physical and mental health of the elderly.

## Figures and Tables

**Table 1:** Pharmacological effects and mechanisms of *A. sinensis* on cardiovascular and cerebrovascular diseases.

Diseases	Pharmacological Effects	Mechanism	References
Cardiovascular diseases	Influence of myocardial ischemic injury	decrease the secretion of inflammatory factors such as NO, IL-6, and TNF-*α*	[25,26,27]
		regulate the My D88/TLR4/NF-κB signaling pathway	
		inhibit the increase of Bcl-2 expression	
		promote the decrease of Bax expression	
	Protection of myocardial cells	down-regulate the expression level of cleaved-caspase-3 and miR-22	[28,29,30,31,32,33]
		increase the expression level of Bcl-2 and miR-4701-3p	
		inhibit the endoplasmic reticulum stress-IRE1/ASK1/JNK signaling pathway	
		promote the proliferation of H9c2 cardiomyocytes	
		inhibit the protein expression of calcineurin and T-type calcium channel subtypes	
		reduce the level of autophagy, and the expression of autophagy-related proteins	
	Inhibition of myocardial infarction and myocardial fibrosis	reduce the release of CK, LDH, MDA, endogenous H_2_O_2_ and ROS in cardiomyocytes, and the expression of Bax and TGF-*β*1 and caspase3	[34,35,36,37,38,39]
		enhance the expression of Bc1-2, GSH-Px and SOD	
		inhibit macrophage infiltration	
		reduce the expression level of pro-inflammatory factors	
		regulate the expression of AMPK-PGC1α pathway	
Cerebrovascular diseases	Influence of cerebral ischemic injury	upregulate the expression levels of the Tnfaip8l2 and Ahsg genes	[40,41,42,43,44,45,46,47,48]
		regulate the expression levels of PI3K/Akt/eNOS proteins	
		increase the activities of SOD and GSH-Px	
		reduce levels of MDA, endogenous H_2_O_2_, and reactive ROS	
	Improvement in vascular dementia	promote the expression of Bcl-2 protein	[49,50,51,52]
		inhibit the expression of Bax protein	
		regulate the PINK1/Parkin pathway	
	Protection of brain cells	down-regulating the expression of GRP78, CHOP, and Caspase-12	[53,54,55]
		reduce the level of LDH in cells, and the apoptosis of PC12 cells	
		Lower the levels of MDA, TNF-*α*, and IL-1*β* in brain tissues, and the Bax levels in the hippocampus	
		increase the activity of SOD and the level of Bcl-2	
Other vascular diseases	Regulation of blood pressure and blood lipids	up-regulate the expression levels of the Tnfaip8l2 and Ahsg genes	[39,56,57,58]
		regulate the expression levels of PI3K/Akt/eNOS proteins	
		increase the activities of SOD and GSH-Px	
		reduce levels of MDA, endogenous H_2_O_2_, and reactive ROS	
	Anti-atherosclerosis	clear excess free radicals in the body	[59,60,61,62,63,64,65]
		increase the activity of antioxidant enzymes, the level of NO	
		reduce plasma endothelin levels, membrane lipid peroxidation	
		regulate MAPKs, TLR4/NF-*κ*B, Nrf2/HO-1, and PI3K/Akt/mTOR signaling pathway	
	Protection of vascular endothelial cells	increase the activity of SOD and NO, endothelial nitric oxide synthase in cells	[66,67]
		alleviate oxidative damage to vascular endothelial cells	
		lower levels of LDH and MDA	
		increase the expression of vascular endothelial growth factor, the proliferation ability of bone marrow-derived mesenchymal stem cells	
